# A Classification Model to Predict the Rate of Decline of Kidney Function

**DOI:** 10.3389/fmed.2017.00097

**Published:** 2017-07-19

**Authors:** Ersoy Subasi, Munevver Mine Subasi, Peter L. Hammer, John Roboz, Victor Anbalagan, Michael S. Lipkowitz

**Affiliations:** ^1^Department of Engineering Systems, Florida Institute of Technology, Melbourne, FL, United States; ^2^Department of Mathematical Sciences, Florida Institute of Technology, Melbourne, FL, United States; ^3^Rutgers Center for Operation Research, Rutgers University, Piscataway, NJ, United States; ^4^Department of Medicine, Icahn School of Medicine at Mount Sinai, New York, NY, United States; ^5^Andor Labs, Morrisville, NC, United States; ^6^Department of Medicine, Georgetown University Medical Center, Washington, DC, United States

**Keywords:** chronic kidney disease, biomarker, proteomics, glomerular filtration rate, proteinuria, combinatorics, Boolean, logical analysis of data

## Abstract

The African American Study of Kidney Disease and Hypertension (AASK), a randomized double-blinded treatment trial, was motivated by the high rate of hypertension-related renal disease in the African-American population and the scarcity of effective therapies. This study describes a pattern-based classification approach to predict the rate of decline of kidney function using surface-enhanced laser desorption ionization/time of flight proteomic data from rapid and slow progressors classified by rate of change in glomerular filtration rate. An accurate classification model consisting of 7 out of 5,751 serum proteomic features is constructed by applying the logical analysis of data (LAD) methodology. On cross-validation by 10-folding, the model was shown to have an accuracy of 80.6 ± 0.11%, sensitivity of 78.4 ± 0.17%, and specificity of 78.5 ± 0.16%. The LAD discriminant is used to identify the patients in different risk groups. The LAD risk scores assigned to 116 AASK patients generated a receiver operating curves curve with AUC 0.899 (CI 0.845–0.953) and outperforms the risk scores assigned by proteinuria, one of the best predictors of chronic kidney disease progression.

## Introduction

Chronic kidney disease (CKD), defined by reduced glomerular filtration rate (GFR), proteinuria, or structural kidney disease, is a worldwide growing public health problem. Many subjects with renal disease of most etiologies progress to renal failure and end-stage renal disease (ESRD) requiring dialysis or renal transplantation, despite the best current therapies (1–7). Identification and characterization of novel biomarkers and targets of therapy for the ESRD patients remains a major focus of the current research in kidney disease and has been the objective of a number of studies, such as the African American Study of Kidney Disease and Hypertension (AASK). AASK examined traditional and non-traditional risk factors for progression of CKD in African-Americans with hypertension and CKD. Data from AASK showed that despite what is still considered optimal therapy of renin–angiotensin system blockade and reduction of systolic blood pressure to less than 130 mmHg, more than half of the patients developed the composite outcome of doubling of Cr, ESRD, or death (7). To date, there has been no better therapy to supplant this strategy. Several possible interventions, including controlling blood pressure (6), treating diabetes (3), modifying dietary protein intake (2), and using medications that might have renoprotective effects (6, 8, 9), have been tested in clinical trials. In all cases, the residual rate of progression of renal disease has remained significant. One approach to addressing this lack of effective therapy is to identify biomarkers of progression of kidney disease, with the hope of identifying new pathways for disease that may be avenues to therapy. One such biomarker may, for example, be the APOL1 gene, as polymorphisms in this gene predict progression of CKD (10). While there are prediction models that can classify the likelihood of progression to ESRD within a defined time period (11), there are no such models for rate of decline of GFR. We therefore performed a pilot study using surface-enhanced laser desorption ionization/time of flight (SELDI-TOF) mass spectrometry to determine whether, by using the novel combinatorics method logical analysis of data (LAD) (6, 8, 9, 12–14), we could detect patterns of protein expression that predict rapid or slow progression on the basis of GFR slope.

## Materials and Methods

### AASK Trial

AASK was a randomized double-blinded treatment trial motivated by the high rate of hypertension-related renal disease in the African-American population and the scarcity of effective therapies (15). The study began as a 21-center randomized double-blinded treatment trial of 1,094 African-Americans patients, aged 18–70 years with hypertension and renal failure with measured GFR between 20 and 65 mL/min/1.73 m^2^ body surface area. Other known causes of renal disease such as diabetes were exclusion criteria as was proteinuria >2.5 g/g creatinine. Patients were randomized to two blood pressure goals and either the angiotensin-converting enzyme inhibitor (ACEi) ramipril, beta blocker metoprolol, or calcium channel blocker amlodipine. The initial AASK results were mixed (6). It was shown that while ACEi therapy can slow the progression of renal disease, there was still a high rate of progression to renal failure. Lower blood pressure did not provide any benefit over usual blood pressure goals. After the clinical trial phase, AASK reenrolled 691 of the surviving patients into the observational AASK Cohort study. In the AASK trial phase, GFR was measured by iothalamate clearance at baseline then every 6 months for up to 5 years (6). Least squares slopes of GFR decline from the 6-month time point until censoring were used to define rapid and slow progressors for this study.

**Figure 1:**
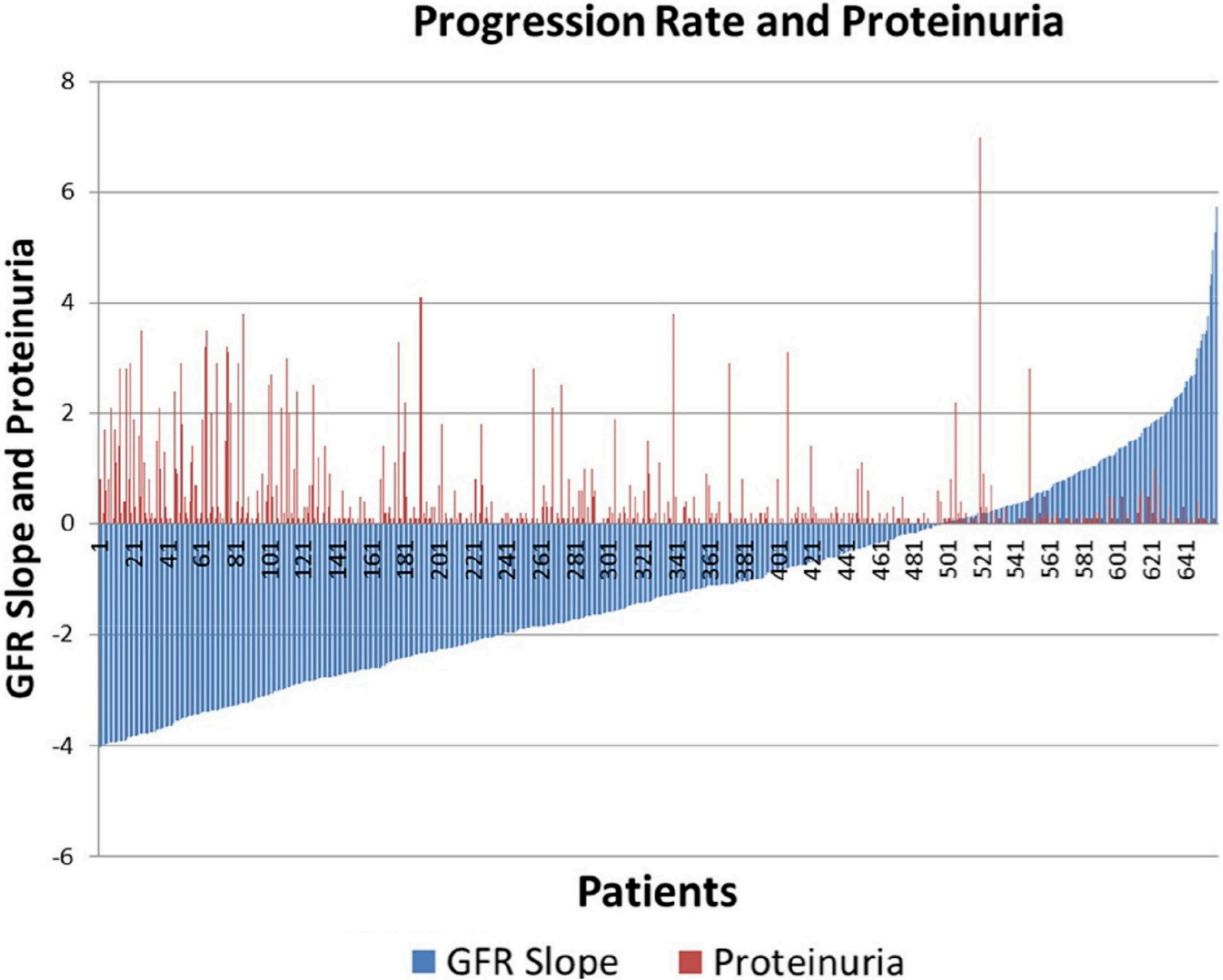
Distribution of glomerular filtration rate (GFR) slope mL/min/year (blue bars) and proteinuria (red bars) in AASK.

There was significant heterogeneity of progression rate of renal disease in the AASK trial as can be seen in Figure 1. The rate of decline of GFR after 6 months in the trial (chronic GFR slope) is depicted in blue for each patient from most rapid decline (negative slope) on the left, to the least rapid (positive slope) on the right of the Figure 1. It is generally assumed that the expected rate of decline of GFR with aging is 1 mL/min/year (16, 17), although longitudinal studies have raised questions about this assumption (18, 19). Using this estimate, approximately 30% of the patients in Figure 1 did not progress (right side, slope >1 mL/min/year), while approximately 30% progressed rapidly (left side, slope <3 mL/min/year). Of interest, it is also apparent that proteinuria, while the strongest predictor of progression rate in most studies, is not an ideal predictor in that there are a number of slow progressors with significant proteinuria (red bars, right), while a significant number of rapid progressors had no or minimal proteinuria (absence of red bars, left).

### Study Subjects

This study was approved by the Institutional Review Board at the Icahn School of Medicine at Mount Sinai with waiver of informed consent, since the AASK patients had consented to the storage and future use of the samples, and the data were deidentified to the Investigators. We have performed a pilot study on a selected subset of AASK subjects based on the GFR slopes of all AASK patients presented in Figure 1. Two sets of subjects were selected from the AASK study: “rapid progressors” (most rapid quartile of GFR slope after 3 months, 57 patients) and “slow progressors” (slowest quartile of GFR slope after 3 months, 59 patients). Patients were matched for blood pressure goal and medication assignment in AASK. Table 1 describes the patient population for this pilot study. Fast progressors were more likely male, had lower measured GFR, and a higher degree of proteinuria. GFR slope was disparate as expected. While GFR was significantly different, the magnitude of the difference was relatively small and would not be expected to dramatically affect the serum proteome. Proteinuria was also very different between the two groups. This is consistent with prior data (20) that showed that proteinuria is the strongest predictor of GFR slope progression in AASK. The discrepancy in gender was fortuitous and unexpected, since gender did not predict GFR slope in AASK (20).

**Table 1:** Patient characteristics.

	Slow (*n* = 59)	Rapid (*n* = 57)	*p*-Value
Glomerular filtration rate (GFR) slope	+2.18 ± 1.13	−6.64 ± 1.38	<0.0001
GFR	53.6 ± 11.6	45.3 ± 12.2	<0.001
Proteinuria	0.10 ± 0.19	1.09 ± 1.37	<0.0001
Age	53.4 ± 11.6	51.2 ± 12.1	NS
BMI	30.0 ± 6.0	32.0 ± 7.4	NS
Male	29	39	<0.001
Female	30	18	

### Sample Preparation

Aliquots of serum from the 1-year visit for each subject were processed in a standardized protocol, used for clinical assays, and then frozen at −80°C. The serum samples from the study subjects were divided into 5–8 μL aliquots, so that sera were not thawed more than twice. After adding internal standard (insulin, 5,734.5 Da), binding buffer (PBS, containing 25 mM imidazole, pH 7), mixing, and centrifuging, 5 μL aliquots were placed on metal chelation ProteinChips (IMAC30) charged with copper and prepared as suggested by the manufacturer (Cyphergen Biosystems, Fremont, CA, USA) using 100 mM CuSO_4_ for 15 min. The energy-absorbing matrix was α-cyano-4-hydroxy-cinnamic acid (sinapinic acid). Samples were analyzed in a random fashion, with both rapid and slow progressors assayed on each 8-spot chip. Samples were analyzed in duplicate. The operators were blinded to the identity of the samples.

### Analysis Using SELDI-TOF-MS

**Figure 2:**
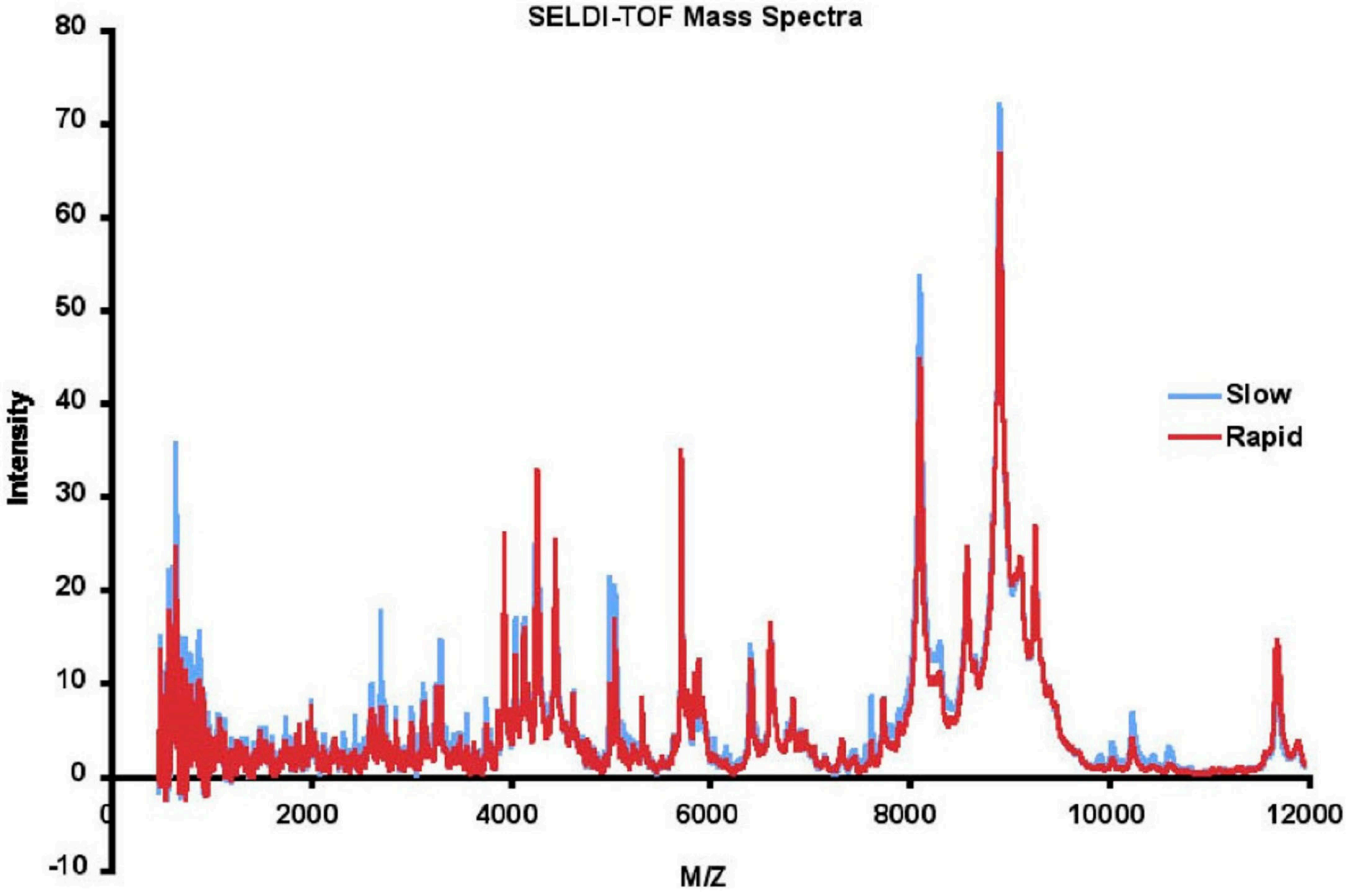
Mean surface-enhanced laser desorption ionization/time of flight (SELDI-TOF) mass spectra of slow (blue) vs rapid (red) progressors.

Masses (range 0–20,000 Da) and intensities were determined using a Protein Biology System 2 SELDI-TOF mass spectrometer (Ciphergen Biosystems, Freemont, CA, USA) and Protein Chip Biomarker software (version 3.0, Ciphergen Biosystems). The laser intensity was adjusted depending on the signal to noise ratio of the mass peak heights to background. The spectra were generated using signal averaging of 90 laser shots. Size and amplitude controls were run daily. A series of proteins with known masses were used as external mass calibrators. Peak intensities were normalized to the internal standard, insulin, 5,734.5 Da. For LAD analysis, SELDI spectra were binned in 2 Da mass unit intervals. The SELDI data were accurate and reproducible. The detected mass for insulin in the 116 samples was 5,737.3 ± 1.1, within 0.06% of the MW of insulin, which is within expected ranges for SELDI. The mean intensity of the insulin peak was 28.96 ± 2.02. All intensities were subsequently normalized using the internal insulin standard before applying data analysis techniques. The results for each patient were highly reproducible, with the mean correlation coefficient between runs for each patient 0.820 ± 0.115. The mean of the SELDI-TOF spectra for 57 rapid and 59 slow progressors is depicted in Figure 2. We eliminated data with *m/z* < 500 as we do not believe peptides with masses this small will be meaningful, and data with *m/z* > 12,000 as the intensities above this value dropped significantly, and accuracy of the SELDI reader at larger masses is limited. This resulted in a dataset with 116 AASK patients (57 rapid and 59 slow progressors) with 5,751 serum proteomic features.

### Application of LAD

In this study, we applied the LAD methodology to build a classification model based on the serum proteomic features for distinguishing the rapid progressors from the slow progressors in AASK dataset. LAD is a two-class classification method based on the theory of Boolean functions, optimization, and combinatorics.

Logical analysis of data is a multistep procedure consisting of (1) discretization, (2) support set selection, (3) pattern generation, (4) classification, and (5) cross-validation. The goal of LAD as applied in this case to rate of progression of CKD is to detect patterns or “combinatorial biomarkers” consisting of restrictions imposed on the values of the intensities of a combination of several masses in the SELDI data. The technique generates patterns nearly exhaustively and in an algorithmically efficient way, and then uses a collection of patterns in a “classification model” that can predict progression rate of the AASK patients selected in this study. In contrast to many statistically based methods, the procedures of LAD are guided by the collective strength of the subsets of features (protein/peptide peaks), without being necessarily restricted to those which have the highest individual correlation coefficients with the outcome.

The initial step of LAD is the transformation of numeric features into binary features without losing predictive power. The procedure consists of finding cutpoints for each numeric feature (13, 21). LAD uses efficient algorithms to “discretize” the SELDI mass intensities by detecting “cutpoints” that discriminate in our case rapid from slow progressors. The discretization step may produce many binary features some of which may be redundant. The LAD method then employs combinatorial optimization algorithms to extract a “support set,” a smallest (irredundant) subset of binary features, which can distinguish every pair of rapid and slow progressors in the SELDI dataset. “Patterns” are the key ingredients of LAD method. The “pattern generation” step uses the binary features in combination to produce a set of rules (combinatorial patterns) which can define homogenous subgroups of interest within the data. The simultaneous use of two or more features allows the identification of more complex rules that can be used for the precise classification of an observation. In the “classification” step, the method uses additional optimization techniques to generate a model consisting of a small number of patterns that can accurately classify patients as rapid or slow progressors. In the final step of LAD, the model’s performance is evaluated through cross-validation experiments.

The LAD results described in the following sections were obtained with the use of “Ladoscope” (22), a publicly available implementation of LAD, written and maintained by Pierre Lemaire. Each of the steps involved in the construction of a LAD classifier (discretization, feature selection, pattern generation, model selection, and classification), as described in this study, are available in Ladoscope software package.

### LAD-Based Risk Scores

**Figure 3:**
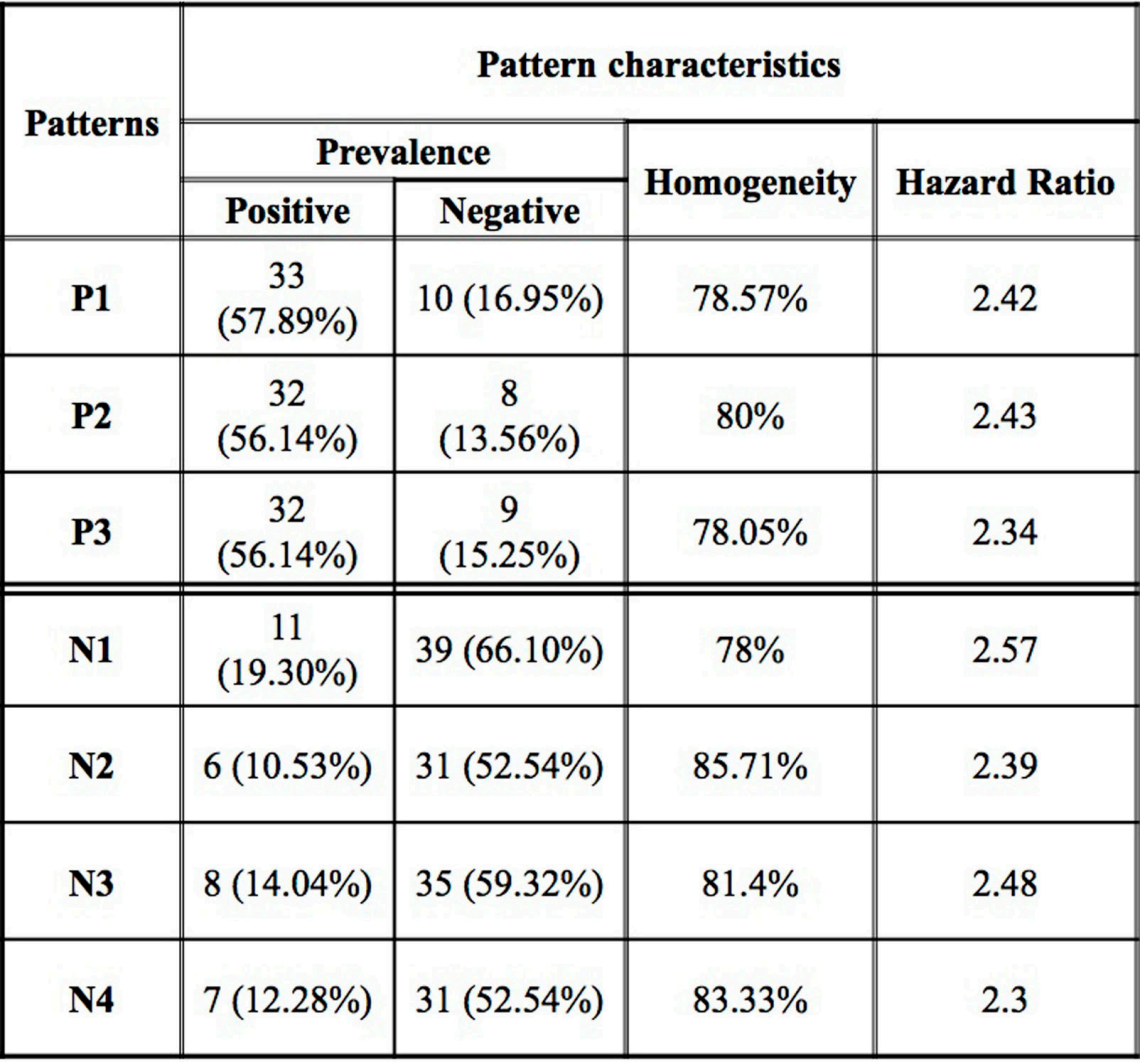
Logical analysis of data pattern characteristics. Prevalence is the proportion of all rapid (slow) progressors covered by the pattern. Homogeneity is the percent of rapid (slow) progressors among all those patients covered by a positive (negative) pattern.

**Figure 4:**
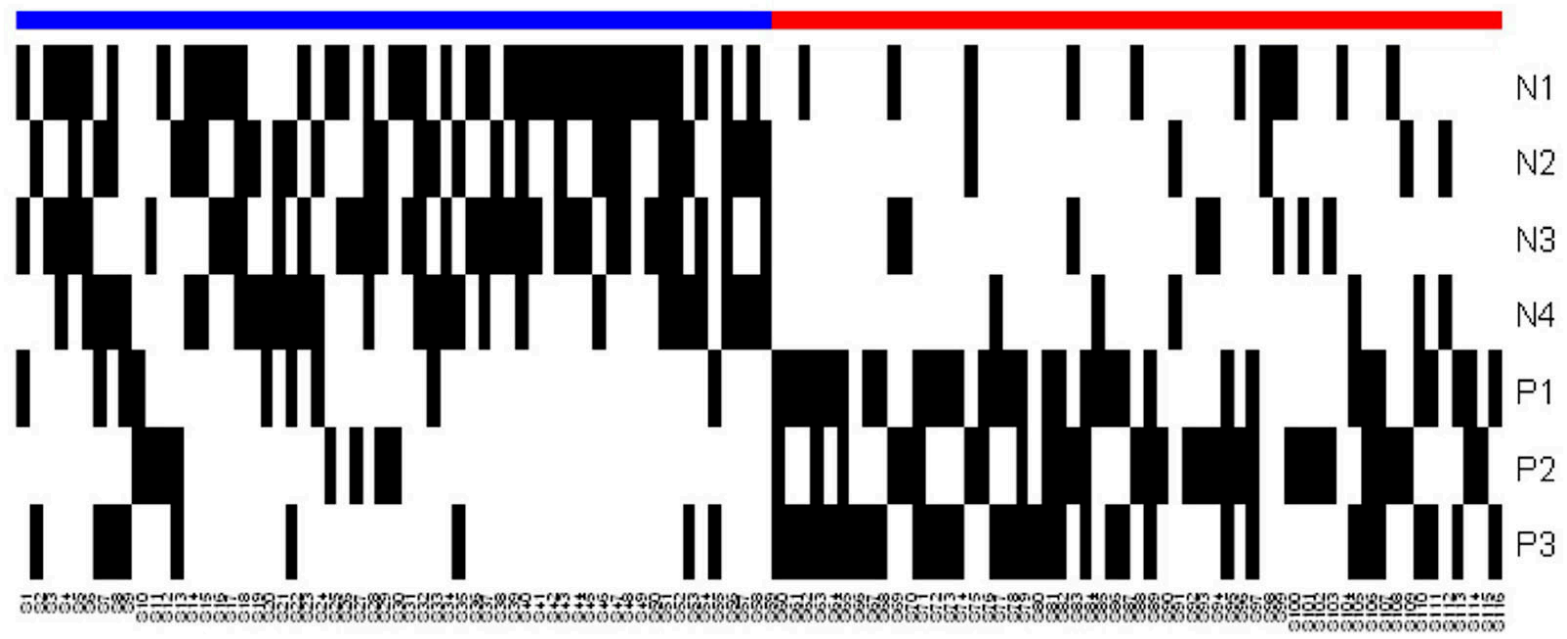
Heatmap of the logical analysis of data model. Blue: slow progressors (negative class), red: fast progressors (positive class), and black: Pattern covers the observation.

An observation whose measurements satisfy the defining conditions of a positive (negative) pattern, but do not satisfy the conditions of any of the negative (positive) patterns, can be easily “classified” as being rapid (slow) progressor. However, as can be seen in Figure 3 and the heatmap in Figure 4, many patients satisfy the defining conditions both of some positive and of some negative patterns. The LAD model ultimately characterizes a patient as a rapid or slow progressor on the basis of a “discriminant” that takes into account all the patterns that cover the patient and employs a simple weighting procedure: if the collection of all positive and negative patterns contains *P* positive and *N* negative patterns, and if a patient π satisfies the defining conditions of *p* of the positive and *n* of the negative patterns, then the discriminant value δ(π) of patient π is defined as δ(π) = (*p/P*) − (*n/N*), i.e., the fraction of positive patterns that cover the patient minus the fraction of negative patterns that cover the patient. The patient is then classified as being rapid or slow progressor, respectively, if the sign of is positive or negative. Note that absolute value δ(π) ≤ 1, and when δ(π) = 0 the observation is unclassified.

The discriminant can also be used as a risk score: the more positive the discriminant, the more likely the patient will be a rapid progressor. In order to generate a positive valued risk score for the AASK patients, we normalized the discriminant values by (δ(π) + 1)*/*2 for all observations in the dataset.

## Results

The main goal of this study is to identify a small set of SELDI-TOF peaks to develop LAD models for the purpose of (i) classifying an individual observation as a rapid or slow progressor based on serum proteomic features and hence, (ii) predicting the progression of CKD. The dataset for the 57 rapid progressors and 59 slow progressors consists of 5,751 intensities at intervals of 2 mass units derived from the SELDI-TOF data.

We observed that a number of peaks differed between rapid and slow progressors (Figure 2). The mean data for rapid and slow progressors were highly reproducible (correlation coefficient > 0.98 between samples for each individual), and of interest, most of the peaks that differed were greater in the slow progressors, suggesting that rapid progression may relate to loss/absence of protective proteins/peptides.

### Preprocessing

The SELDI-TOF data collected for this study contained many peaks, each of which potentially corresponds to the intensity level of a specific protein. In fact, many of these peaks are irrelevant for the recognition of a rapid progressor as opposed to a slow progressor. In order to obtain a classification model effectively and efficiently, we applied a preprocessing procedure to retain only those relevant peaks distinguishing between rapid and slow progressors.

First, we applied the LAD method to generate high quality combinatorial patterns with characteristics (1) degree 1—a single feature is used in the definition of the pattern; (2) homogeneity of at least 80%—the proportion of rapid (slow) progressors among all those patients covered by the pattern; and (3) prevalence of at least 80%—the proportion of rapid (slow) progressors covered by the pattern. We then retained the peaks participating in only these patterns. After the application of this filtering procedure, we obtained a subset of 135 relevant peaks. Next, we applied LAD method on the preprocessed data to generate higher degree LAD patterns to form an accurate classification model. In this set of 135 expression levels, we derived a minimal support set utilizing the intensities at 7 masses as listed in Table 2, which allows us to accurately distinguish the slow progressors from the rapid progressors. The statistical correlations of the selected features with GFR slope are also shown in the table.

**Table 2:** Support set of surface-enhanced laser desorption ionization/time of flight masses whose intensities are used to create the logical analysis of data classification model.

SELDI mass (*m*/*z*)	Correlation coefficient	Correlation rank
2,018	0.039	4,115
2,756	0.260	16
2,780	0.252	28
5,266	0.065	3,290
9,940	0.194	348
11,274	0.133	1,565
11,752	0.192	378

It can be seen that many of the intensities correlate very poorly with slope. If all 5,751 features were ordered according to their decreasing correlations with the outcome (in absolute value), the attribute at *m/z* 2,756 would appear as the feature with the correlation rank 16 (16th best correlation coefficient), but *m/z* 2,018 would have rank 4,115 (see the last column of Table 2). We emphasize these facts in order to point out that selecting the features only based on high correlations with the outcome may not necessarily lead to discovery of those which may have a significant predictive power when combined with other features.

### Classification Model

**Figure 5:**
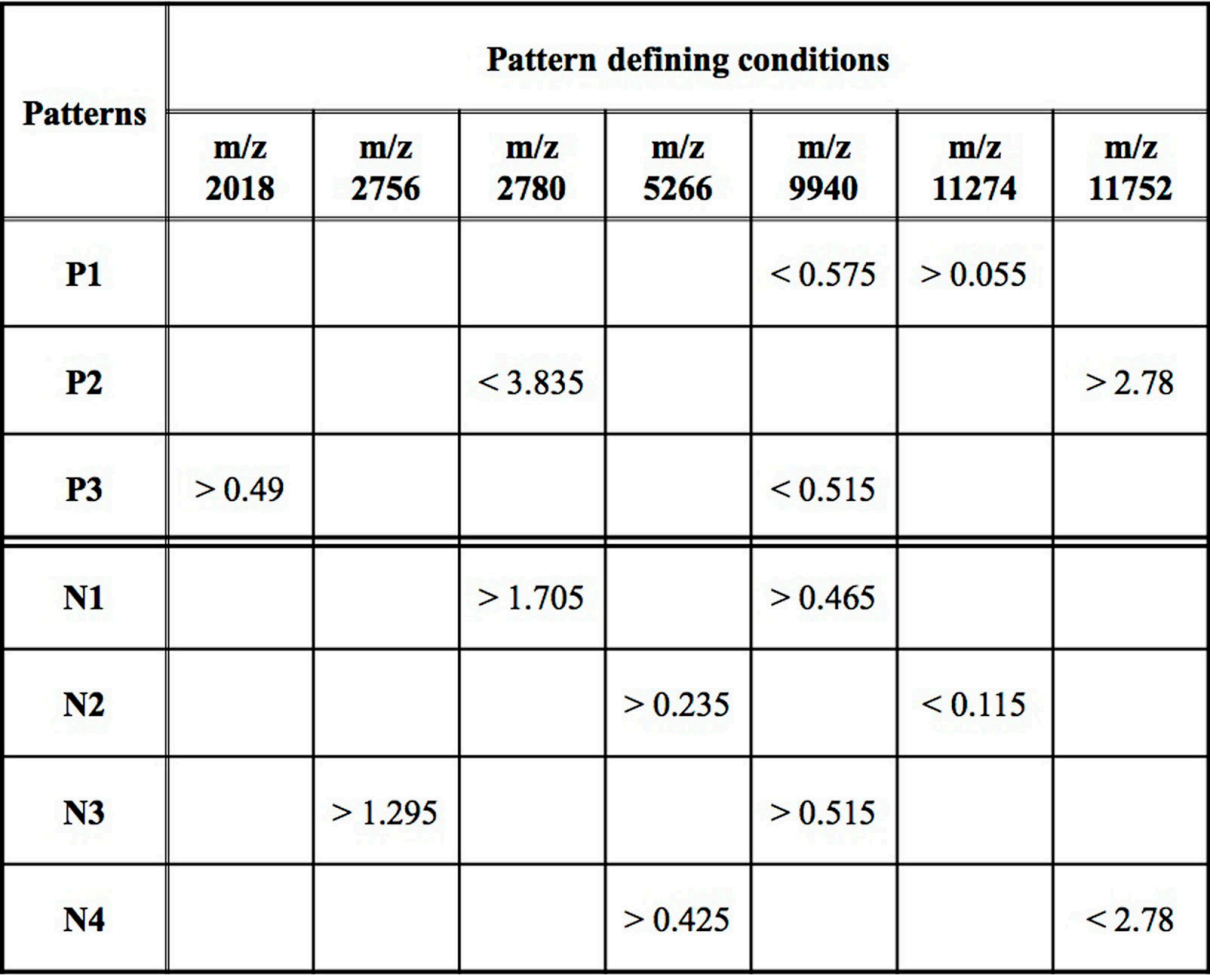
Logical analysis of data model for predicting rapid vs slow progression in surface-enhanced laser desorption ionization/time of flight AASK data. Positive patterns (*P*) predict rapid progression and negative patterns (*N*) slow progression. Mass/charge ratio (*m*/*z*) is a measure of protein mass; data represent peak intensity at the individual masses. A patient’s sample fulfills fast progression positive pattern 1 (*P*1) if the intensity of the peak at *m/z* 9,940 < 0.575 and the intensity at *m/z* 11,274 > 0.055. Other patterns are interpreted in a similar fashion.

While an individual feature can partially predict an observation of being a rapid or slow progressor, the simultaneous use of two or more features allows for the definition of more complex rules (combinatorial biomarker) that can be used for the precise classification of an observation. We used LAD to classify rapid and slow progressors using the seven masses in Table 2 to generate patterns comprised intensities at two masses each. This final LAD classification model consists of three positive patterns (predicting rapid progression) and four negative patterns (predicting slow progression) as presented in Figure 5. The pattern characteristics including prevalence, homogeneity, and hazard ratio of each pattern are shown in Figure 3.

The patterns involved in the final classification model are high quality patterns since each pattern has degree two (i.e., its definition involves at most two peaks) and small degree patterns are easy to interpret; patterns are of high prevalence (each pattern covers most observations from the respected class) on average positive patterns cover about 57% of the rapid progressors and negative patterns cover 58% of the slow progressors; and patterns had high homogeneity (each positive (negative) pattern covers mostly rapid (slow) progressors and only a few slow (rapid) progressors) and on average 79% of the observations covered by positive patterns are rapid progressors and 82% of the observations covered by negative patterns are slow progressors (Figure 3).

The overlapping black regions in the heatmap (Figure 4) imply that an observation is covered by more than one pattern, and hence, only a few patterns are sufficient to separate the rapid and slow progressors.

### Validation of the LAD Model

The performance of the final LAD classification model presented in Figure 5 was evaluated through *k*-folding (10-folding in this case) cross-validation technique: the SELDI-TOF data are randomly partitioned into *k* = 10 approximately equal parts; one of these subsets is designated as “test set,” a model is built on the remaining nine subsets which form the “training dataset,” and then tested by classifying the cases in the test set using the model. This procedure is repeated *k* = 10 times, always taking another 1 of the 10 parts in the role of the test set; the data set is then rerandomized into 10 new subsets, and the procedure repeated 9 additional times for a total of 100 tests. The average accuracy (proportion of correctly classified observations), sensitivity (proportion of correctly classified rapid progressors), and specificity (proportion of correctly classified slow progressors) are then reported as a quality measure of the proposed model in Table 3.

**Table 3:** Cross-validation of the logical analysis of data classification model.

Accuracy (%)	Sensitivity (%)	Specificity (%)	Hazard ratio
80.6 ± 0.11	78.4 ± 0.17	78.5 ± 0.16	2.72

As can be seen, the LAD model predicts the rate of decline of kidney function among AASK samples with high sensitivity and specificity and accuracy.

### LAD-Based Risk Scores

Table 4 depicts the risk scores assigned the AASK samples as well as the proportion of the rapid progressors in each quintile of patients based on the discriminant of the SELDI positive and negative patterns. As can be seen in Table 4, all of the patients in the highest and lowest risk groups were classified correctly by the LAD classification model and the risk scores are very close to the proportion of rapid progressors in each risk group.

**Table 4:** Logical analysis of data risk scores from lowest risk of progression (Group 1) to highest (Group 5).

Risk group	# of observations	% Rapid progressors	Average risk score
1	23	0.00	0.087
2	23	21.74	0.275
3	23	47.83	0.498
4	23	73.91	0.697
5	24	100.00	0.924

**Figure 6:**
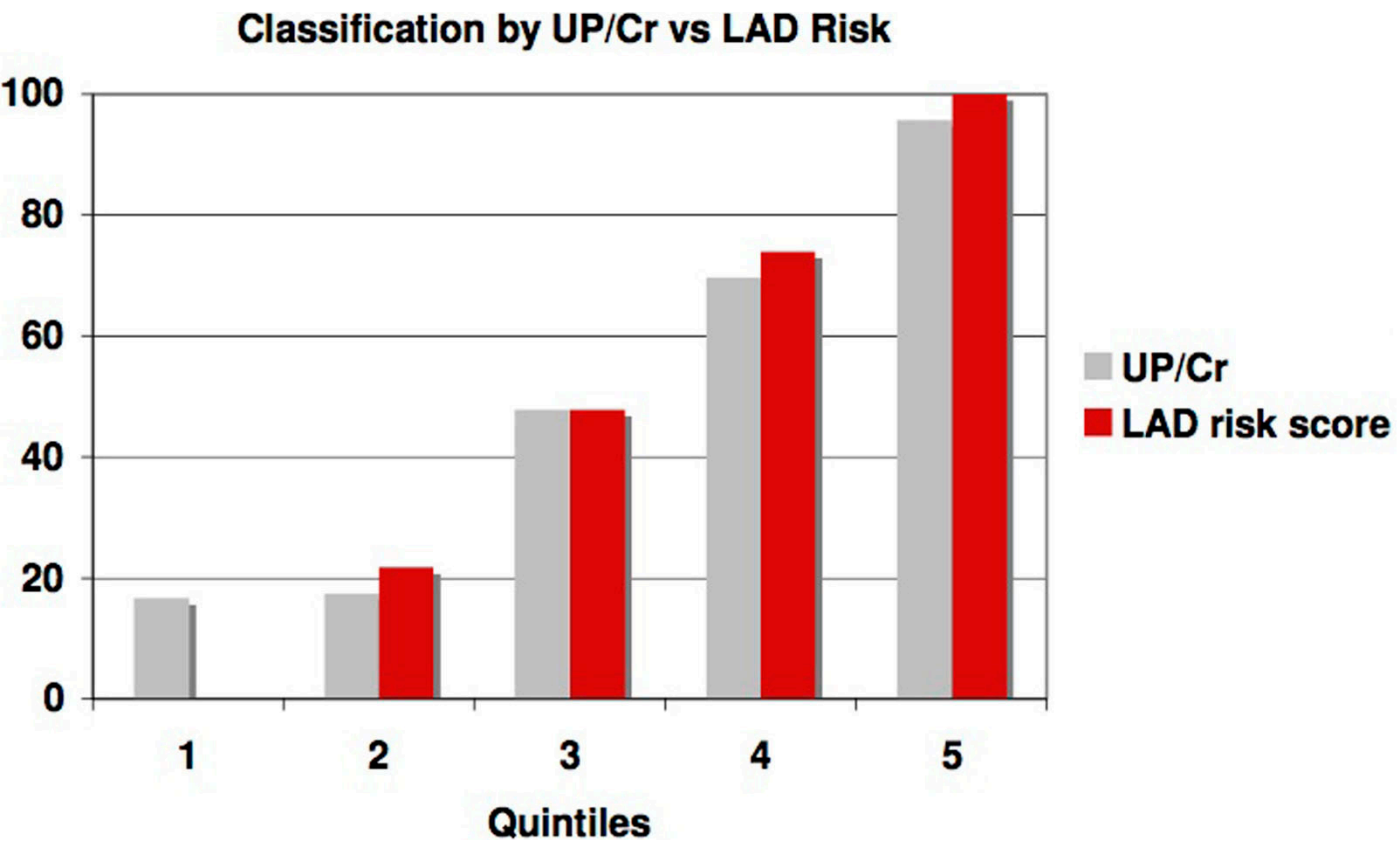
Proportion of patients classified as rapid progressors either by proteinuria (gray bars) or logical analysis of data (LAD) risk score (red bars) in each risk quintile.

This significantly outperforms a similar classification of patients using proteinuria, the strongest traditional predictor of risk of progression in AASK, especially in identifying slow progressors. Table 5 and Figure 6 show that the high levels of proteinuria accurately classify rapid progressors, misclassifying only a few patients, but the lowest proteinuria risk group misclassified 17% of the rapid progressors. Furthermore, the level of proteinuria in risk groups 1–3 varied from a UP/Cr ratio of 0.02–0.08 (probably on the order of 30–100 mg/day), levels that in the clinic would likely be indistinguishable due to testing error and day-to-day variability. Since 45% of patients in risk group 3 are rapid progressors even levels of proteinuria that are considered normal may not define a low risk group.

**Table 5:** Urine protein/urine creatinine risk scores.

Risk group	# of observations	% Rapid progressors	Average UP/UCr
1	23	16.7	0.02
2	23	17.4	0.03
3	23	47.8	0.08
4	23	69.67	0.31
5	24	95.7	1.35

We also generated receiver operating curves as a measure of the effectiveness of the LAD discriminant at predicting GFR slope. The area under the curve is 0.899 (confidence interval 0.845–0.953), which is highly significantly different from 0.5 (a value that would be found for a non-predictive test). A similar analysis using urine protein/creatinine ratio was also predictive but less robust (the area under the curve is 0.845 and confidence interval (0.774–0.916)), similar to the comparison in Figure 6.

## Discussion

In this study, we present an unbiased pattern-based classification approach to predict the rate of decline of kidney function in CKD using a SELDI-TOF proteomics data set. We construct a classification model by applying the LAD methodology to the mass peak profiles of 57 rapid progressors and 59 slow progressors in AASK mass spectra data containing 5,751 serum proteomic features. The classification model has an accuracy of 80.6% obtained through 10 times 10-folding cross-validation experiments. Despite significant predictive power of the LAD classification model, it contains 3 positive patterns (describing rapid progression) and 4 negative patterns (describing slow progression) and the patterns involved in the model are extremely simple (each only involves two features in pattern description) and use only 7 out of 5,751 SELDI peaks. The LAD discriminant is used as a risk score and effectively identifies the patients in different risk groups as defined by GFR slope. Thus, we have shown that applying LAD to a large proteomics data set can generate relatively simple models that predict CKD progression. As shown in Table 2, the components of the model, in this case protein peaks, do not have to correlate with outcomes individually. Previous studies have shown that LAD can also be applied to large clinical data sets with similar results-only a few of large numbers of exposures can strongly predict clinical outcomes.

Extracting unbiased meaningful information from a large-scale data such as AASK requires the integration of sophisticated mathematical techniques and efficient computerized algorithms. Several studies have applied existing algorithms for pattern matching to SELDI datasets (23–25), but no significant attempts to optimize such methods have been attempted.

Logical analysis of data was shown to outperform previously employed methods on publicly available datasets (20, 26–30). First introduced by Crama et al. (12), LAD has been successfully applied in various areas of science and technology. The medical applications of LAD (13, 26–33) demonstrate clearly that the method provides excellent solutions both for the analysis of medical problems using clinical datasets and for that of multiparameter datasets generated in the fields of genomics [such as gene expression microarrays (28, 29)] and proteomics [such as mass spectrometry (27–29)]. The results of LAD applied to such problems turned out not only to be extremely accurate but also to yield new kinds of tools both for direct applications, such as diagnosis and prognosis, and for biomedical research about the importance of targeting particular combinations of genes and proteins for therapeutic purposes.

Among other specific features of LAD which support its usefulness in biomedical informatics, LAD performs an unbiased exhaustive examination of the entire set of clinical, genomic, or proteomic features, without *a priori* excluding those data which have either low statistical correlations with the outcome, or low expression levels. A novel and essential feature of LAD is its ability to discover not only potential biomarkers but also potential “combinatorial biomarkers” (combinations of features) and its exhaustive search for underlying combinatorial patterns.

The ability to exhaustively examine a large number of potentially predictive factors such as the 5,751 protein peaks in this study and generate a model that has very few components (7 protein peaks) should allow investigators to focus on only a small number of clinical factors or protein pathways to try to better understand and treat complex diseases such as renal failure. In the particular case of SELDI proteomics, the exact identification of the proteins in the peaks can be difficult and the ability to focus on a limited number of peaks is critical.

Identifying both progressors and non-progressors in groups of patients at risk for ESRD in itself is very important, in that it will allow us to focus efforts on those at greatest risk using the best current therapies. We may also be able to avoid complications of therapy if we can identify a truly low risk group that can just be observed rather than treated (note for example that that BP control to a lower than usual goal (<130*/*80) in AASK took almost four BP drugs, each with its own side effect profile). If an LAD risk score is associated with less than a 10% risk of progression, patients in that category could likely be observed rather than immediately treated with aggressive therapy or could forego such therapy if side complications arise. While current therapies for CKD are relatively benign, it should be noted that recent clinical trials have been performed with other drugs that are likely to have much more aggressive side effect profiles. If such medications are effective at slowing CKD progression, robust predictors of disease progression would be very helpful evaluating the overall risk/benefit ratio of therapy, which may not be beneficial at low progression risk.

The strengths of this proposal are the well-defined slopes derived from multiple, measured GFR points over a significant 5-year length of time, and the ability with LAD to derive a predictive model with a small number of patterns and peaks in an unbiased fashion. Limitations of the study include the small sample size allowed by this pilot study using the most rapid and slowest progressors, and the fact that the mass resolution of SELDI-TOF technology does not allow direct protein identification from the resulting spectra.

The ultimate goal of a future project motivated by this study is the identification of new protein targets that may suggest the new therapies that are desperately needed for CKD. The ability of LAD to identify a small number of protein peaks that predict outcomes makes it feasible to perform further studies to identify the individual proteins in those peaks. This pilot study has shown that the combination of mass spectrometry and LAD analysis of the data should allow an approach to finding biomarkers for GFR slope and potential for discovery of new disease pathways that are unique to that from other methods and could also potentially generate mass spectrometry marker panels for progression risk.

## Ethics Statement

This study was carried out in accordance with the recommendations of institutional Review Board of the Icahn School of Medicine at Mount Sinai with waiver of informed consent. All AASK subjects gave written informed consent in accordance with the Declaration of Helsinki to store serum samples for use in future studies; the waiver was granted because the current study used deidentified stored samples.

## Author Contributions

ML helped design and supervised the entire project, contributed to data acquisition and analysis, and participated in writing the manuscript. ES and MS performed the combinatorial analysis and participated in writing the manuscript. JR designed and supervised the mass spectrometry studies and reviewed and edited that paper. VA performed the mass spectrometry and reviewed and edited the paper. PH participated in the study design and combinatorial analysis.

## Conflict of Interest Statement

The authors declare that the research was conducted in the absence of any commercial or financial relationships that could be construed as a potential conflict of interest. The handling Editor declared a shared affiliation, though no other collaboration, with one of the authors, MSL and states that the process nevertheless met the standards of a fair and objective review.
